# Baseline characteristics of study sites and women enrolled in a three-arm cluster randomized controlled trial: PMTCT uptake and retention (pure) Malawi

**DOI:** 10.1186/s12978-017-0343-0

**Published:** 2017-07-11

**Authors:** Monique van Lettow, Hannock Tweya, Nora E. Rosenberg, Clement Trapence, Virginia Kayoyo, Florence Kasende, Blessings Kaunda, Mina C. Hosseinipour, Michael Eliya, Fabian Cataldo, Salem Gugsa, Sam Phiri, for the PURE Malawi consortium, Sam Phiri, Hannock Tweya, Salem Gugsa, Clement Trapence, Saulos Mhlanga, Frank Chimbwandira, Michael Eliya, Mina Hosseinipour, Nora E. Rosenberg, Innocent Mofolo, Virginia Kayoyo, Christopher Stanley, Monique van Lettow, Fabian Cataldo, Misheck Nkhata, Florence Kasende, Megan Landes, Don Mathanga, Atupele Kapito-Tembo, Blessings Kaunda-Khangamwa, Levison Chiwaula, Gowokani Chirwa, Erik Schouten, Veena Sampathkumar

**Affiliations:** 1 Dignitas International, Zomba, Malawi; 2 Dalla Lana School of Public Health, University of Toronto, Toronto, Canada; 3 Lighthouse Trust, Lilongwe, Malawi; 4 The International Union against Tuberculosis and Lung Disease, Paris, France; 5 University of North Carolina Project, Lilongwe, Malawi; 6 Department of Epidemiology, University of North Carolina, Chapel Hill, NC USA; 7 University of Malawi, College of Medicine, Malaria Alert Centre, Blantyre, Malawi; 8 Department of Medicine, University of North Carolina School of Medicine, Chapel Hill, USA; 9 Department of HIV and AIDS, Ministry of Health, Lilongwe, Malawi; 10 Department of Public Health, University of Malawi, College of Medicine, School of Public Health and Family Medicine, Lilongwe, Malawi

**Keywords:** PMTCT, Uptake, Retention, Peer-support, Cluster randomized trial, Malawi, Feasibility

## Abstract

**Background:**

Malawi introduced an ambitious public health program known as “Option B+” which provides all HIV-infected pregnant and breastfeeding women with lifelong combination antiretroviral therapy, regardless of WHO clinical stage or CD4 cell count. The PMTCT Uptake and REtention (PURE) study aimed at evaluating the effect of peer-support on care-seeking and retention in care.

**Methods/design:**

PURE Malawi was a three-arm cluster randomized controlled trial that compared facility-based and community-based models of peer support to standard of care under Option B+ strategy. Each arm was expected to enroll a minimum of 360 women with a total minimum sample size of 1080 participants. 21 sites (clusters) were selected for inclusion in the study. This paper describes the site selection, recruitment, enrollment process and baseline characteristics of study sites and women enrolled in the trial.

**Results:**

Study implementation was managed by 3 partner organizations; each responsible for 7 study sites. The trial was conducted in the South East, South West, and Central West zones of Malawi, the zones where the implementing partners operate. Study sites included 2 district hospitals, 2 mission hospitals, 2 rural hospitals, 13 health centers and 1 private clinic. Enrollment occurred from November 2013 to November 2014, over a median period of 31 weeks (range 17–51) by site. A total of 1269 HIV-infected pregnant (1094) and breastfeeding (175) women, who were eligible to initiate ART under Option B+, were enrolled. Each site reached or surpassed the minimum sample size. Comparing the number of women enrolled versus antenatal cohort reports, sites recruited a median of 90% (IQR 75–100) of eligible reported women. In the majority of sites the ratio of pregnant and lactating women enrolled in the study was similar to the ratio of reported pregnant and lactating women starting ART in the same sites. The median age of all women was 27 (IQR 22–31) years. All women have ≥20 months of possible follow-up time; 96% ≥ 2 years (24–32 months).

**Conclusion:**

The PURE Malawi study showed that 3 implementing partner organizations could successfully recruit a complex cohort of pregnant and lactating women across 3 geographical zones in Malawi within a reasonable timeline.

**Trial registration:**

This study is registered at clinicaltrials.gov - ID Number NCT02005835. Registered 4 December, 2013.

## Plain english summary

Malawi introduced a new strategy that aims at preventing HIV infection from mother to child. There is no cure for HIV, but medication (antiretroviral treatment) is available to slow the progression of the virus and help prevent transmission of HIV to others. The so called “Option B+” strategy means that all HIV-infected pregnant and breastfeeding women start antiretroviral treatment as soon as they are diagnosed. The PMTCT Uptake and REtention (PURE) study evaluated whether support provided by peers (mothers who are HIV-infected and on treatment themselves) can help to increase the number of HIV-infected pregnant and breastfeeding women to start treatment (uptake) and keep them in care and on treatment (retention). The study took place in 21 health facilities, where mothers either received no peer support (7 sites), peer support in the facility (7 sites) or peer support in the community (7 sites). This article describes how the study was successfully set up and managed by 3 organizations. Between November 2013 and November 2014, a total of 1269 HIV-infected women were included in the study in the 21 health facilities. We describe that it is possible to set up a complex study across 3 geographical zones in Malawi.

## Background

In 2011, Malawi introduced an ambitious public health strategy known as “Option B+”, providing a standardized lifelong combination antiretroviral therapy (ART) regimen to all HIV-infected pregnant and breastfeeding women, irrespective of their CD4 cell count or World Health Organisation (WHO) clinical stage of HIV infection. This public health approach for the prevention of mother to child transmission (PMTCT) of HIV has since been adopted by several other countries in the region and was included in the updated WHO guidelines in 2013 [1]. Initial data have demonstrated that the Option B+ strategy has resulted in large increases in the number of pregnant women accessing PMTCT [2]. However, concerns have been raised about the level of attrition of women from care [3]. Although the strategy offers an attractive rapid ART scale-up and has the potential to profoundly impact maternal and infant outcomes, services supporting the implementation of Option B + have not been formally evaluated. Several issues were identified at the national level as potential threats to the successful implementation and scale up of the strategy: a) potential suboptimal uptake of ART by asymptomatic pregnant women due to low treatment literacy and stigma; b) low adherence to ART and poor follow-up of HIV exposed infants; and c) lack of psycho-social support for long term retention in this relatively asymptomatic patient population [4]. Hence, it seemed apparent that the identification of interventions to support PMTCT uptake and retention would be the first step towards improvement. A recently published systematic review identified 9 completed studies and 5 ongoing trials which examined initiation of ART in pregnant women [5]. While the authors report several promising interventions for improving ART initiation, the quality of evidence was insufficient to support recommendations. In addition, results for ART initiation in pregnant women were not independently examined, and maternal retention in PMTCT care and exposed infants care were not assessed.

Although randomized controlled trials are promoted as the highest level of evidence for the evaluation of interventions, the implementation of rigorous trials within the context of the public health sector are complex and therefore still uncommon [6]. PURE Malawi embraced the challenge of implementing a multi-site controlled trial within the context of public health clinics under real world conditions in Malawi. PURE Malawi was a three-arm cluster randomized controlled trial that aimed at evaluating whether facility- or community-based peer-support would enhance care-seeking and retention in care by HIV-infected pregnant and breastfeeding women, their HIV-exposed infants, and their male sex partners, and ultimately improve health outcomes in all three populations. The hypothesis was that enhanced support for women and their families within facilities and/or community would result in improved retention in the continuum of PMTCT care. The primary trial outcome is the proportion of women retained in care, 2 years after they initiated antiretroviral therapy under the Option B+ strategy. This paper describes the site selection, recruitment, enrollment process and baseline characteristics of study sites and women enrolled in the trial.

## Methods

PURE Malawi was a three-arm stratified cluster randomized controlled trial that compared facility-based and community-based models of peer support to standard of care under Option B+. The three arms differed with respect to the presence and location of one-on-one patient education and support, support groups, visit reminders, and missed visit follow-up. The trial was unblinded to study participants and health care providers, but blinded to outcome assessors. A comprehensive description of the study arms was reported elsewhere [7]. The PURE trial was managed by the PURE Malawi consortium comprising of governmental, non-governmental, research, and academic partners. The consortium was led by Lighthouse Trust. Dignitas International, The University of Malawi College of Medicine Malaria Alert Centre, and University of North Carolina Project were each responsible for trial implementation in one Malawian zone. The trial was conducted in the South East, South West, and Central West zones of Malawi, the zones where the implementing partners operate. Selection and responsibilities of all consortium members were described elsewhere [7].

### Site selection and recruitment targets

Clusters (i.e. health facilities) were the unit of randomization in this trial. Public health facilities in the South East, South West, and Central West health zones of Malawi, covering the central and southern geographical regions of Malawi, were assessed for eligibility in the trial. Sites were eligible if they provided Option B+ services, did not have additional PMTCT interventions or research activities beyond the Malawi national standard of care, and were expected to have at least 20 eligible women for Option B+ in a 6 month period. HIV care in Malawi is free and standardized in all public health facilities. Site assessments, selection and randomization of the sites were described elsewhere [7].

Twenty-one selected sites were stratified into 3 groups of 7, with each group having sites of comparable size and expected retention, based on Ministry of Health historic data. Depending on the size of the health facility, each site was expected to reach a minimum enrollment target of 20, 50, 75, or 125 pregnant or breastfeeding women eligible for the Option B+ program. Each arm was expected to enroll a minimum of 360 women with a total minimum sample size of 1080 participants. Sites could exceed this target if they finished enrollment prior to a 6 month period.

### Enrolment process and index participants

Prior to study implementation, all Ministry of Health staff working at ANC and ART departments in the selected sites received training in study arm specific standard operating procedures, adherence to ethical standards and management of data collection tools (Good Clinical Practice). At each site, a PURE site coordinator was selected to oversee the study implementation.

All newly identified HIV positive women identified during pregnancy, delivery, or breastfeeding while seeking antenatal care (ANC) or initiating ART services at the study sites were eligible for inclusion in the study. Women consented to participate and be followed in the site where they had sought ANC or ART services and were explained in which arm the site was allocated.

Based on existing PMTCT monitoring data, we anticipated 6 months until sample size was achieved.

All participating women were assigned a unique study ID. Study ID stickers were placed in the study register, the mother’s existing health passport and on all routine clinic records for data extraction and tracking.

The index participants were newly confirmed HIV-infected pregnant and breastfeeding women; eligible to initiate ART under Option B+, in the 21 selected health facilities. To attain the primary trial outcome, enrolled women were expected to be followed until the end of the study period (31st July 2016), transfer out, default, or death.

### Data collection and management during recruitment period

All newly identified HIV positive women were eligible for inclusion in the study. Therefore, routinely collected health facility cohort data prepared during quarterly supervision visits [8] was used to compare the number of women enrolled in the study with the number of eligible women seeking care at the health facility during the recruitment period. Monthly ANC cohort data included the number of women newly registered, newly diagnosed with HIV or already known HIV-infected, and those already on ART. ART cohort data included the number of pregnant and lactating women having started ART per quarter.

Facility characteristics data, including availability of HIV Testing Services (HTS), ANC and ART services were collected by existing study personnel throughout the enrolment period.

During study enrollment, health facility staff used PURE registration and PURE enrolment forms to collect data on mothers’ age, expected delivery date (pregnant women) or date of birth of the child (lactating women), marital status, and whether the partner lives in the catchment area.

At the ART initiation visit, a routinely used clinical card was initiated and treatment initiation data were recorded, including date, age and reason for initiation of ART (WHO stage or pregnant/lactating), TB and KS. The data collection and analytic methods, including a detailed analysis plan, for the final trial outcomes were described elsewhere [7].

### Compensation of health facility for study specific activities

During the enrolment period, each health facility received 1000 MK (equivalent to approximately 2 USD) per correctly enrolled study participant, in addition to a median amount of 5000 MK per month (equivalent to approximately 10 USD) for correct organization and storage of clinical and study records as stipulated by the national guidelines and PURE protocols. This amount was shared among the facility staff involved in the study.

## Results

PURE Malawi sites included 2 district hospitals, 2 mission hospitals, 2 rural hospitals, 13 health centers and 1 private clinic. Table 1 shows PURE Malawi sites by health zone, implementing partner and study arm. Each implementing partner managed the implementation of the study across the 7 sites in the respective health zone where the partners operate. The organization Mothers2Mothers provided additional support for training and implementation in all Arm 2 sites. Table 2 shows the number of Ministry of Health staff trained by site and cadre prior to study implementation. Enrollment initiation varied per trial site from November 2013 through January 2014. Recruitment was completed in November 2014. Median enrollment time per site was 31 weeks, ranging from 17 to 51 weeks. Table 3 describes the number of pregnant and lactating women enrolled per site and arm, in addition to the number of eligible women reported in cohort reports at ANC and ART during the recruitment period. Each site and arm reached or surpassed the minimum sample. However, a few participants (4%) were enrolled later than the desired end date of July 31st 2014, making 2 years of follow up impossible by July 31, 2016, the trial end date.

**Table 1:** PURE Malawi sites by health zone, implementing partner and study arm

Implementing partner	Central west zone	South east zone	South west zone
	University of North Carolina Project, Lilongwe	Dignitas International, Zomba	Malaria Alert Centre, Blantyre
Arm 1	Daeyang Luke Hospital Public^c^	Mulanje District Hospital^a^	Mwanza District Hospital^a^
	Kasinje Health Centre^d^	Mimosa Dispensary^e^	Chirimba Health Centre^d^
	Dr David Livingstone Memorial Clinic^f^		
Arm 2	Lobi Rural Hospital^c^	Muloza Health Centre^d^	Makhetha Health Centre^d^
		Mbiza Health Centre^d^	Masenjere Health Centre^d^
			Mdeka Health Centre^d^
			Kadidi Health Center^d^
Arm 3	Biriwiri Health Centre^d^	Mulanje Mission Hospital^b^	Trinity Mission Hospital^b^
	Nsipe Health Centre^d^	Mulomba Health Centre^d^	
	Bilira Health Center^d^	Mpala Health Centre^d^	

^a^District Hospital

^b^Mission Hospital

^c^Rural Hospital

^d^Health Centre

^e^Dispensary

^f^Private clinic

**Table 2:** Number of facility staff trained in study procedures and good clinical practices

Arm and facility name	ART/PMTC coordinators	ART/PMTCT providers	HTC providers	ART clerks	Total
Arm 1: Standard					
Mulanje District Hospital	3	7	3	2	15
Daeyang Luke Hospital Public	2	7	3	1	13
Mwanza District Hospital		1		2	3
Mimosa Dispensary		2	2	2	6
Kasinje Health Centre		4	2	2	8
Dr. David Livingstone Memorial Clinic		6		1	7
Chirimba Health Centre	2	6		2	8
Arm 2: Facility Support					
Muloza Health Centre		3	4	2	9
Makhetha Health Centre	1	13	1		15
Mbiza Health Centre	1	4	2	2	9
Masenjere Health Centre			2	4	6
Mdeka Health Centre	4	6	2	2	14
Lobi Rural Hospital		3	2		5
Kadidi Health Centre		7			7
Arm 3: Community Support					
Mulanje Mission Hospital	1	8	3	2	14
Trinity Mission Hospital			5	10	15
Mulomba Health Centre		2	4	1	7
Mpala health Centre	1	3	2	2	8
Biriwiri Health Centre		3	3	2	8
Nsipe Health Centre		4	2	1	7
Bilira Health Center		3	2	2	7

**Table 3:** PURE study enrollment per site and arm versus eligible women reported in cohort data from the same facilities

PURE recruitement							MoH program report cohort data					
Arm and facility name		Target per site	Total enrolled	Enrollment period	Pregnant women enrolled	Lactating women enrolled	MoH program report period ANC DATA (monthly)	New ANC registrations	HIV positives women^a^ (not on ART) in ANC	MoH program report period ART DATA (quarterly)	Pregnant women started ART	Lactating women started ART
Arm 1: Standard												
Mulanje District Hospital		125	127	23Jan’14-29Aug’14	122	5	01Jan’14-01Sep’14	1631	149	01Jan’14-30Sep’14	191	34
Daeyang Luke Hospital Public		75	75	24Dec’13-04Sep’14	65	11	01Dec’13-01Oct’14	1298	71	01Oct’13-30Sep’14	111	18
Mwanza District Hospital		50	74	09Jan’14-25aug’14	67	8	01Jan’14-01Sep’14	2248	54	01Jan’14-30Sep’14	107	26
Mimosa Dispensary		50	52	09Jan’14-10Jul’14	37	15	01Jan’14-01Aug’14	557	61	01Jan’14-30Sep’14	53	28
Kasinje Health Centre		20	49	16Dec’13-08Aug’14	44	5	01Dec’13-1Sep’14	1398	42	01Jan’14-30Sep’14	55	11
Dr. David Livingstone Memorial Clinic		20	20	19Nov’13-17Nov’14	19	1	01Nov’13-01Dec’14	262	21	01Oct’13-31Dec’14	29	1
Chirimba Health Centre		20	50	08Jan’14-17Jul’14	48	2	01Jan’14-01Aug’14	724	66	01Jan’14-30Sep’14	71	18
Total		360	447		402	47		8118	464		617	136
Arm 2: Facility Support												
Muloza Health Centre		125	125	27Nov’13-04Jun’14	108	17	01Nov’13-01Jul‘14	1325	161	01Oct’13-30Jun’14	178	34
Makhetha Health Centre		75	78	13Jan’14-20Aug’14	62	16	01Jan’14-01 Sep’14	711	31	01Jan’14-30 Sep’14	109	39
Mbiza health Centre		50	52	17Dec’13-08Jul’14	48	4	01Dec’13-01Aug’14	598	51	01Oct’13-30Sep’14	112	12
Masenjere Health Centre		50	51	11Dec’13-23Jun’14	43	7	01Dec’13-01Jul’14	754	42	01Oct’13-30Jun’14	67	10
Mdeka Health centre		20	40	06Jan’14-26Jun’14	34	7	01Jan’14-01Jul’14	695	32	01Jan’14-30June’14	46	11
Lobi Rural Hospital		20	40	22Nov’13-31Jul’14	26	14	01Nov’13-01Aug’14	1472	33	01Oct’13-30Sep’14	39	18
Kadidi Health Center		20	42	07Jan‘14-06May’14	40	2	01Jan’14-01Jun’14	527	53	01Jan’14-30June’14	66	2
Total		360	428		361	67		6082	403		617	126
Arm 3: Community Support												
Mulanje Mission Hospital		125	125	16Jan’14-13Oct’14	109	16	01J an’ 14-01Nov’14	1912	265	01Jan’14-31Dec’14	255	41
Trinity Mission Hospital		75	77	04Dec’13-03Oct’14	68	9	01Dec’13-01Nov’14	1529	76	01Oct’13-31Dec’14	119	41
Mulomba Health Centre		50	52	04De c’13-25Jun’ 14	44	8	01Dec’13-01Jul’14	716	23	01Oct’13-30Jun’14	61	20
Mpala health Centre		50	52	18Dec’13-11Jul’14	44	8	01Dec’13-01Aug’14	1650	111	01Oct’13-30Sep’14	165	48
Biriwiri Health Centre		20	26	19Nov’13-24Jul’14	16	10	01Nov’13-01Aug’14	568	21	01Oct’13-30Sep’14	37	20
Nsipe Health Centre		20	31	07Jan’14-28Jul’14	26	5	01Jan’14-01Aug’14	503	29	01Jan’14-30Sep’14	24	9
Bilira Health Center		20	31	02Dec’13-31Jul’14	26	5	01Dec’13-01Aug’14	751	22	01Oct’13-30Sep’14	39	13
Total		360	394		333	61		7629	547		700	192
TOTAL		1080	1269		1096	175		21,829	1414		1934	454

^a^Previous positives (not yet on ART) + new positives

A total of 1269 women were enrolled, 1094 (86%) pregnant and 175 (14%) lactating women.

When comparing number of enrolled pregnant women per site with facility cohort data; 7 sites recruited >100% of the total reported cohort of HIV-infected pregnant women at ANC in the corresponding months. The additional number of pregnant women may have started ART, but not attended ANC in the specific study site. Nine sites recruited >90%, 10 sites 60–90%, and 2 sites 40%–59% of the total reported cohort of pregnant women at ANC in the corresponding months. When comparing the number of women enrolled versus ANC reports, sites recruited a median of 90% (IQR 75–100) of eligible reported women from ANC.

Following quarterly ART cohort report data; in all study sites, a total of 1934 (81%) pregnant and 454 (19%) lactating women had started ART during corresponding quarters. This proportion of pregnant and lactating women was comparable to our study sample.

### Baseline characteristics of study sites

Table 4 shows the characteristic of study sites, with regards to services available during the enrolment period. During the enrolment period, 9 facilities experienced a median of 2 weeks of HIV test kit stock outs (range 2–8) and 13 facilities were not able to provide HTC for a median of 4 weeks (range 1–8) due to stock outs or unavailability of HTC providers. The 6 largest facilities provided ANC and ART services during all week days; in all other facilities ANC and ART services were provided at a median of 3 days (range 2–4) and 1 day (range 1–4) per week, respectively. In 19 of the 21 facilities, ART provision was available at ANC.

**Table 4:** Facility characteristics during enrollment period

Arm and facility name	Enrolment duration in weeks	HIV test kit stockout in weeks	HTC service NOT available in weeks	Number of days per week ANC provided	Number of days per week ART provided	ART provided at ANC (Yes/No)
Arm 1: Standard						
Mulanje District Hospital	31	-	-	5	5	Yes
Daeyang Luke Hospital Public	36	-	-	4	5	Yes
Mwanza District Hospital	33	2	2	5	5	Yes
Mimosa Dispensary	26	-	4	2	1	Yes
Kasinje Health Centre	33	-	1	5	4	Yes
Dr. David Livingstone Memorial Clinic	51	-	4	5	5	No
Chirimba Health Centre	27	-	-	4	1	Yes
Arm 2: Facility Support						
Muloza Health Centre	27	4	4	3	1	Yes
Makhetha Health Centre	31	8	8	3	1	No
Mbiza health Centre	29	-	-	2	1	Yes
Masenjere Health Centre	27	8	8	2	2	Yes
Mdeka Health centre	24	2	2	2	1	Yes
Lobi Rural Hospital	36	-	-	5	5	Yes
Kadidi Health Center	17	2	2	2	1	Yes
Arm 3: Community Support						
Mulanje Mission Hospital	38	-	-	5	5	Yes
Trinity Mission Hospital	43	4	4	5	5	Yes
Mulomba Health Centre	29	-	4	2	1	Yes
Mpala health Centre	29	-	-	3	1	Yes
Biriwiri Health Centre	35	2	3	3	1	Yes
Nsipe Health Centre	29	-	-	3	1	Yes
Bilira Health Center	34	2	2	3	1	Yes

### Baseline characteristics of women enrolled in the trial

Cumulative baseline characteristics of women enrolled in the trial are shown in Table 5.

**Table 5:** Cumulative Baseline characteristics of women included in the study

	Total	Percent	Pregnant	Percent	Lactating	Percent	*P*- value (Chi-square)
N	1269		1094	86%	175	14%	
Health Zone							
Central West	272	21%	223	20%	49	28%	0.07
South East	585	46%	512	47%	73	42%	
South West	412	32%	359	33%	53	30%	
Age							
15–21	266	21%	224	20%	42	24%	0.30
22–28	488	38%	419	38%	69	39%	
29–35	406	32%	355	32%	51	29%	
> 36	103	8%	94	9%	9	5%	
Missing	6	0.5%	2	0.2%	4	2%	
Months pre- or postnatal at enrolment							
< 3 months			370	34%	42	24%	
3–6 months			582	53%	22	13%	
> 6 months			47	4%	51	29%	
Missing			97	9%	61	35%	
WHO Stage at ART initiation							
Stage 1 (asymptomatic)	1211	95%	1044	95%	167	95%	0.12
Stage 2	13	1%	13	1%			
Stage 3	10	1%	7	1%	3	2%	
Stage 4	-		-		-		
Missing	35	3%	30	3%	5	3%	
Marital Status							
Never married	21	2%	16	1%	5	3%	0.001
Maried Monogamous	1068	84%	942	86%	124	71%	
Maried Polygamous	110	9%	96	9%	13	7%	
Divorced	49	4%	27	2%	22	13%	
Widowed	17	1%	7	1%	10	6%	
Missing	7	1%	6	1%	1	1%	
Currently having a sexual partner							
Yes	1193	94%	1053	96%	140	80%	0.001
No	60	5%	29	3%	31	18%	
Missing	16	r 1%	12	r 1%	4	2%	
Current sexual partner living in catchment area							
Yes	994	83%	878	83%	116	83%	0.43
No	133	11%	118	11%	14	10%	
Missing	66	6%	57	5%	10	7%	
Enrollment period							
01Oct-31Dec’13	108	9%	94	9%	14	8%	0.67
01lan-31Mar’14	551	43%	468	43%	83	47%	
01Apr-30Jun’14	417	33%	362	33%	55	31%	
01lul -30Sep’14	186	15%	163	15%	23	13%	
01Oct-31Dec’14	7	1%	7	1%			
Follow up time from Enrolment to Study End							
20–23 months	47	4%	40	4%	7	4%	0.37
24–27 months	467	37%	411	38%	56	32%	
28–32 months	755	59%	643	59%	112	64%	

The median age of all women was 27 (IQR 22–31) years, with no difference between those pregnant and lactating. Pregnant women were enrolled at a median of 4 months (IQR 2–5) prenatal; breastfeeding women at a median of 5 months (IQR 1–11) postnatal. Reason for ART initiation for all women was being pregnant or lactating (regardless of CD4 or WHO stage). Among those with WHO stage reported at ART initiation, 98% of both pregnant and lactating women were reported clinically asymptomatic. There was 1 documented TB case (within the last 2 years) and 1 documented KS case at ART initiation.

Proportionally more lactating women reported being divorced, widowed, or having no sexual partner than pregnant women. Among women with at least one sexual partner, 11% were reported not living in the same catchment area as their partner. There was no difference in recruitment by Health Zone period or the time for follow-up between pregnant and lactating women. The largest proportion of women was recruited between January and July 2014, enabling 96% of women to be followed up for at least 2 years (24–32 months) as part of the trial.

## Discussion

This paper shows that the PURE consortium was successful in recruiting a complex cohort of pregnant and lactating women across 3 geographical zones in Malawi. Each site and trial arm reached or surpassed the minimum required sample size, and the majority of sites recruited over 90% of the reported eligible women attending ANC during the recruitment period. Although cluster designs are often avoided due to the challenges of enrolling at multiple sites within a reasonable timeline, we show that, despite the absence of remunerated study staff within study site, only few participants were enrolled later than the desired end date; allowing for 96% of the cohort to be followed for at least 2 years. We report that there were HIV test kit stock outs or unavailability of the HTC providers, which may have prolonged the enrolment period. Shortages and stock out of commodities at health facilities is a reality in Malawi, however, national guidelines are set up to improve the supply management [8]. In the majority of participating sites, the ratio of pregnant and lactating women enrolled in the study was similar to the ratio of pregnant and lactating women initiated on ART in the same sites. There was no difference in age between the enrolled pregnant and lactating women. One fifth of the women enrolled were young mothers (15–21), which is consistent with national evaluations that describe women in Malawi’s Option B+ program [3]. Proportionally more lactating women reported being divorced, widowed or having no sexual partner. As male partner support is thought to be an important determinant for uptake and outcomes of PMTCT initiatives [9–11], follow up of our cohort of mothers with and without partners will add to the evidence on the impact of male partner involvement approaches on PMTCT services. According to Malawi’s demographic health survey, 14% of Malawian women are married to a man with more than one wife. Polygamy is most common in the Northern Region [12], which may explain why we found a lower proportion of women in polygamous marriage (9%). In our study, 98% of pregnant and lactating women were reported clinically asymptomatic. This high proportion of women starting ART in the earliest stages is an indication of the success of Malawi’s PMTCT programming.

Malawi, as the first country to implement Option B+, has the potential to be a leader in developing and documenting highly effective models of service delivery to pregnant and lactating women and enhance retention in lifelong care and treatment. Cluster randomized controlled trials are needed to determine whether promising interventions are indeed effective. However, the implementation of such trials pose a substantial challenge, especially across multiple regions, different facility types, and several implementing partners. Thus, the ability to select 21 comparable clinics, stratify them in a meaningful way, and have each enroll its target sample size is an achievement. This achievement stems from clear leadership, small incentives to clinical staff, and well-coordinated teams.

This paper described the processes for site selection, recruitment, and enrollment of study participants, along with baseline characteristics of study sites and women enrolled in the trial. Follow-up of participants was completed on July 31st, 2016. The final results and outcomes (per protocol) of this study are reported elsewhere [13]. Alongside this trial, a qualitative study has been implemented to explore experiences of patients and health care workers in relation to the implementation of Option B+ and the trial interventions [14].

### Limitations

We were not able to report the precise number of women who refused to participate in the study, as this data was not collected consistently across the sites. However, compared to facility cohort data, it appears that we were able to enroll the majority of eligible women.

## Conclusions

Malawi, as the first country to implement Option B+, has the potential to be a leader in developing and documenting highly effective models of service delivery to provide needed support to women and enhance retention in lifelong HIV care and treatment approaches. The PURE trial is expected to play a critical role in this process. We show that a consortium of organizations was successful in recruiting a complex cohort of pregnant and lactating women across 3 geographical zones in Malawi within a reasonable timeline.
